# Simultaneous comparison of depth of sedation performance between SedLine and BIS during general anesthesia using custom passive interface hardware: study protocol for a prospective, non-blinded, non-randomized trial

**DOI:** 10.1186/s12871-021-01326-5

**Published:** 2021-04-06

**Authors:** James Harvey Jones, Vinay Ravikumar Nittur, Neal Fleming, Richard L. Applegate

**Affiliations:** 1 Department of Anesthesiology and Pain Medicine, University of California Davis Medical Center, 4150 V Street, PSSB Suite 1200, Sacramento, CA 95817 USA; 2 School of Medicine, University of California Davis, Sacramento, CA USA

**Keywords:** Bispectral index (BIS), Patient state index (PSI), SedLine, Processed electroencephalogram (EEG), Depth of anesthesia, Depth of sedation, Brain function monitoring, Burst suppression state, Electromyography (EMG)

## Abstract

**Background:**

Intraoperative brain function monitoring with processed electroencephalogram (EEG) indices, such as the bispectral index (BIS) and patient state index (PSI), may improve characterization of the depth of sedation or anesthesia when compared to conventional physiologic monitors, such as heart rate and blood pressure. However, the clinical assessment of anesthetic depth may not always agree with available processed EEG indices. To concurrently compare the performance of BIS and SedLine monitors, we present a data collection system using shared individual generic sensors connected to a custom-built passive interface box.

**Methods:**

This prospective, non-blinded, non-randomized study will enroll 100 adult American Society of Anesthesiologists (ASA) class I-III patients presenting for elective procedures requiring general anesthesia. BIS and SedLine electrodes will be placed preoperatively according to manufacturer recommendations and their respective indices tracked throughout anesthesia induction, maintenance and emergence. The concordance between processed EEG indices and clinical assessments of anesthesia depth will be analyzed with chi-square and kappa statistic.

**Discussion:**

Prior studies comparing brain function monitoring devices have applied both sensors on the forehead of study subjects simultaneously. With limited space and common sensor locations between devices, it is not possible to place both commercial sensor arrays according to the manufacturer’s recommendations, thus compromising the validity of these comparisons. This trial utilizes a custom interface allowing signals from sensors to be shared between BIS and SedLine monitors to provide an accurate comparison. Our results will also characterize the degree of agreement between processed EEG indices and clinical assessments of anesthetic depth as determined by the anesthesiologists’ interpretations of acute changes in blood pressure and heart rate as well as the administration, or change to the continuous delivery, of medications at these timepoints. Patient factors (such as burst suppression state or low power EEG conditions from aging brain), surgical conditions (such as use of electrocautery), artifacts (such as electromyography), and anesthesia medications and doses (such as end-tidal concentration of volatile anesthetic or hypnotic infusion dose) that lead to lack of agreement will be explored as well.

**Trial registration:**

Clinical Trials (ClinicalTrials.gov), NCT03865316. Registered on 4 February 2019 – retrospectively registered. Sponsor: Masimo Corporation.

## Background

At present, the end target organ of action for anesthetic agents – the brain – is not routinely monitored given the unclear correlations of brain function monitoring devices in all clinical settings, uncertain benefits in preventing accidental awareness under general anesthesia, variable responses to individual anesthetic agents, and the impact of artifacts [1–3]. Analyses of raw electroencephalogram (EEG) data have characterized anesthetic induction, maintenance, and emergence with specific waveforms and patterns [4]. However, each anesthetic medication produces a unique, but complex, signature on the EEG that must be considered in real-time [5, 6]. At present, routine EEG interpretation during general anesthesia remains impractical given lack of standardized training in this competency and variable support in the medical literature.

Anesthetic overdose, as indicated by burst suppression of the EEG, has been associated with postoperative delirium and increased mortality [7–9]. Anesthetic underdose may risk accidental awareness during general anesthesia and subsequent long-term morbidity, including posttraumatic stress disorder [10–12]. The ideal end-tidal concentration of volatile anesthetic or hypnotic infusion dose for a patient under general anesthesia is dynamic and depends on innumerable patient factors, medications, and clinical situations. Because a complete anesthetic includes amnesia, analgesia, and akinesia, quantifying anesthetic depth is a convoluted, multifactorial estimation. Acute changes, or no changes, in blood pressure and heart rate in response to noxious stimuli currently serve as the primary guide to intraoperative anesthetic management even though vital signs are known to be unreliable indicators of anesthetic depth and are commonly influenced by analgesia and intraoperative events [13].

EEG waveforms can be summarized and translated into dimensionless values over time with proprietary algorithms to offer a simplified, continuous scale of consciousness, thus presenting an additional tool for clinicians to guide attempts to quantify anesthetic depth without the need to interpret raw EEG data [14]. While remaining blind to the mathematical methods or Fourier transform on which the proprietary algorithms are based, clinicians can analyze these processed EEG indices in response to various medications and clinical situations in real-time. Differences in proprietary algorithms between devices may yield disagreements during electromyography (EMG), electrocautery, low power EEG conditions from aging brain, or across anesthetic depth ranges. At present, no individual brain function monitoring device has been shown to be substantially superior.

The Bispectral index (BIS) monitor was the first depth of anesthesia device to analyze the phase and power of EEG frequency bands through a proprietary algorithm, thus distinguishing it from competitors [15, 16]. In contrast, the more recently introduced SedLine monitor provides a patient state index (PSI) to quantify anesthetic depth by analyzing the spatial and temporal gradients of EEG frequency bands in the anterior-posterior dimension [17]. The accuracy and clinical utility of these two monitors depend on various factors including EMG, electrocautery, patient age, and pre-existing neurological disorders [18–21]. Therefore, despite their appeal, processed EEG data must be interpreted with caution.

Prior investigators have attempted to simultaneously compare the performance of BIS and PSI by placing multiple sensors on subjects’ foreheads [22, 23]. However, with limited space and common sensor locations between the two devices, it is not possible to place both commercial sensor arrays according to the manufacturer’s recommendations. Non-standard lead placement compromises the interpretation of the comparisons thus made. We hypothesized that processed EEG indices from BIS and SedLine monitors can be measured simultaneously using individual electrode placement and a custom designed interface box to combine and split signals, thus allowing performance to be compared across a broad range of anesthetic depths. This data collection system allows us to investigate the concordance between these monitors and real-time clinical assessments of anesthetic depth and analyze the actions of the anesthesiologists in response to acute changes in blood pressure and heart rate.

## Methods

### Study design and setting

This data collection system will be used in a prospective, non-randomized, non-blinded trial approved by the Institutional Review Board (IRB) at the University of California, Davis. The anesthesia providers responsible for the intraoperative care of included patients will be blinded to the processed EEG indices and not permitted to utilize these indices to guide the anesthetic management. Any adverse events observed throughout the trial will be communicated with the IRB and sponsor.

### Study population

Written informed consent will be obtained from all study participants or their surrogates. We plan to enroll up to 100 patients in this study to observe whether or not the custom built passive interface hardware allows for accurate, simultaneous comparison of BIS and SedLine devices. Because of the absence of any comparative data, this sample size was arbitrarily chosen so that the study participants will include a heterogenous group of surgeries, comorbid conditions, and patient demographics. All patients will meet the following criteria:

#### Inclusion criteria

- Patients aged 18 years or older
- American Society of Anesthesiologists’ (ASA) status I-III
- English-speaking
- Scheduled for surgical or non-surgical procedures requiring general anesthesia

#### Exclusion criteria

- Any deformities or devices that may prevent application of EEG sensors to the patient’s forehead
- Developmental delay
- Other conditions for which the patient is otherwise deemed not suitable for the study at the discretion of the investigator. Unsuitable conditions include inadequate forehead space to allow for all electrodes to be properly positioned. Patients will also be excluded based on chronic medical conditions that may impact EEG recordings (such as a history of frontal sinus surgery or epilepsy).
- Surgeries in the lateral or prone position, which may alter the impedance of the electrodes and make it difficult for the investigators to troubleshoot.

### Study devices

The following devices will be utilized in this protocol:

- FDA-cleared Root™ Rainbow Technology Multi-Function Docking Station (Masimo Corporation)
- FDA-cleared SedLine patient module (Masimo Corporation)
- FDA-cleared BIS system (Medtronic/Covidien)
- FDA-cleared 3rd party EEG/ECG sensors
- Laptop computer with PulseOX Automatic Data Collection (ADC) and Rugloop (data collection software)
- Custom built passive interface box to facilitate simultaneous connection of BIS and SedLine monitors; this interface has no active electronics

### Processed EEG setup

**Fig. 1:**
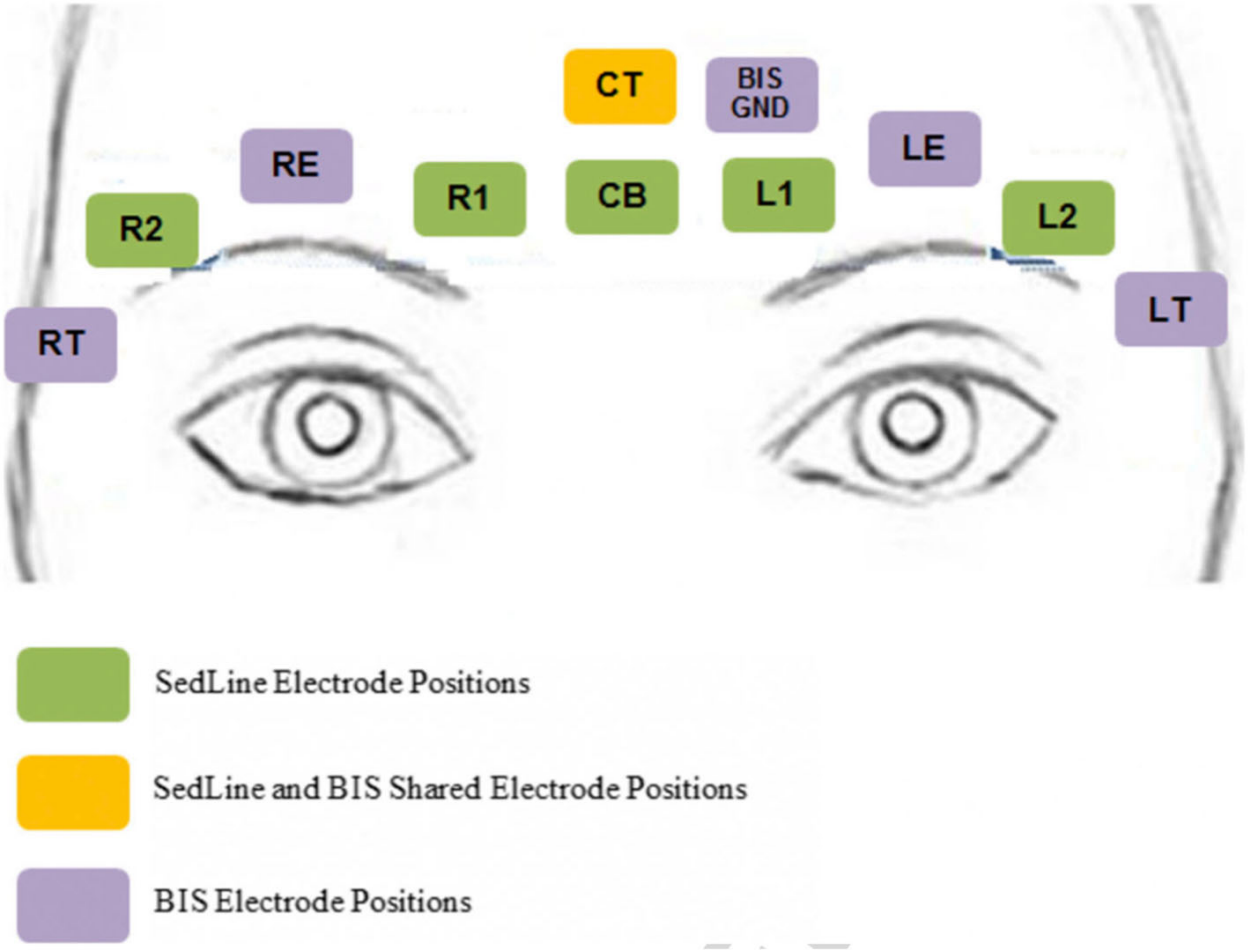
Proper placement of 11 EEG electrodes to allow for simultaneous recording of BIS and PSI indices

After the sub-investigator, principal investigator, research coordinator, or other participating site staff obtains written informed consent from included participants on the day of surgery, the preoperative baseline heart rate and blood pressure will be collected. The patient’s age, weight, race, ethnicity, and significant co-morbid conditions will be recorded on a case report form (CRF). The forehead skin of included patients will be prepared with 70% alcohol wipes to decrease impedance for 11 third-party EEG sensors (Kendall 1050NPSM Neonatal ECG Electrodes). BIS ground channel (BIS GND) and sensor electrodes will be placed in locations according to the manufacturer’s recommendations and labelled right temple (RT), right eye (RE), left temple (LT), and left eye (LE). SedLine sensor electrodes will be similarly placed according to the manufacturer’s instructions and include 4 active channels (R1, R2, L1, and L2) and 1 ground channel (CB) [24]. The BIS and SedLine electrode arrays share an additional common reference channel (CT). The final locations of these EEG sensors are shown in Fig. 1.

**Fig. 2:**
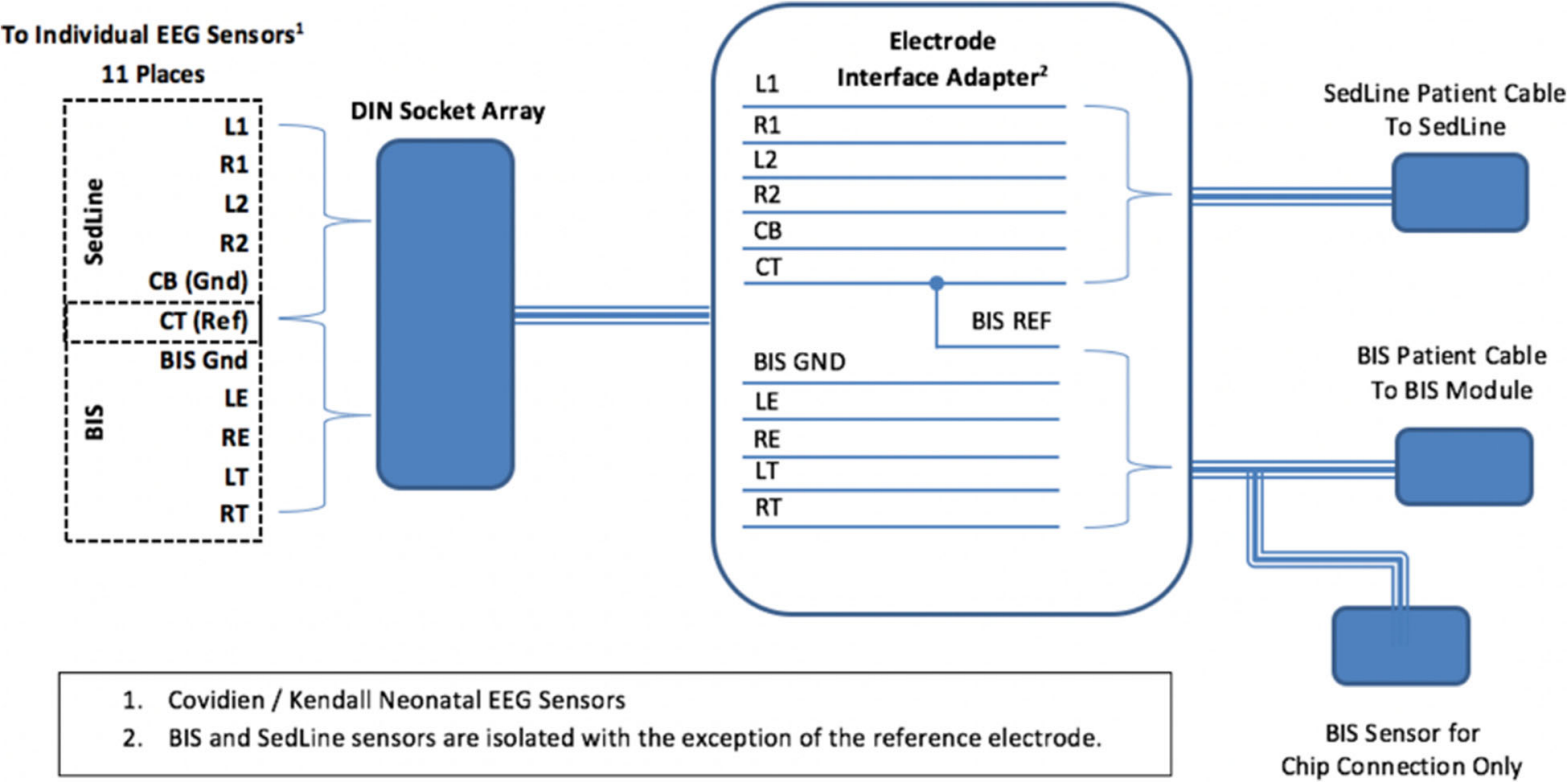
Custom interface adapter to allow for concurrent connection of EEG sensors to both BIS and SedLine modules

**Fig. 3:**
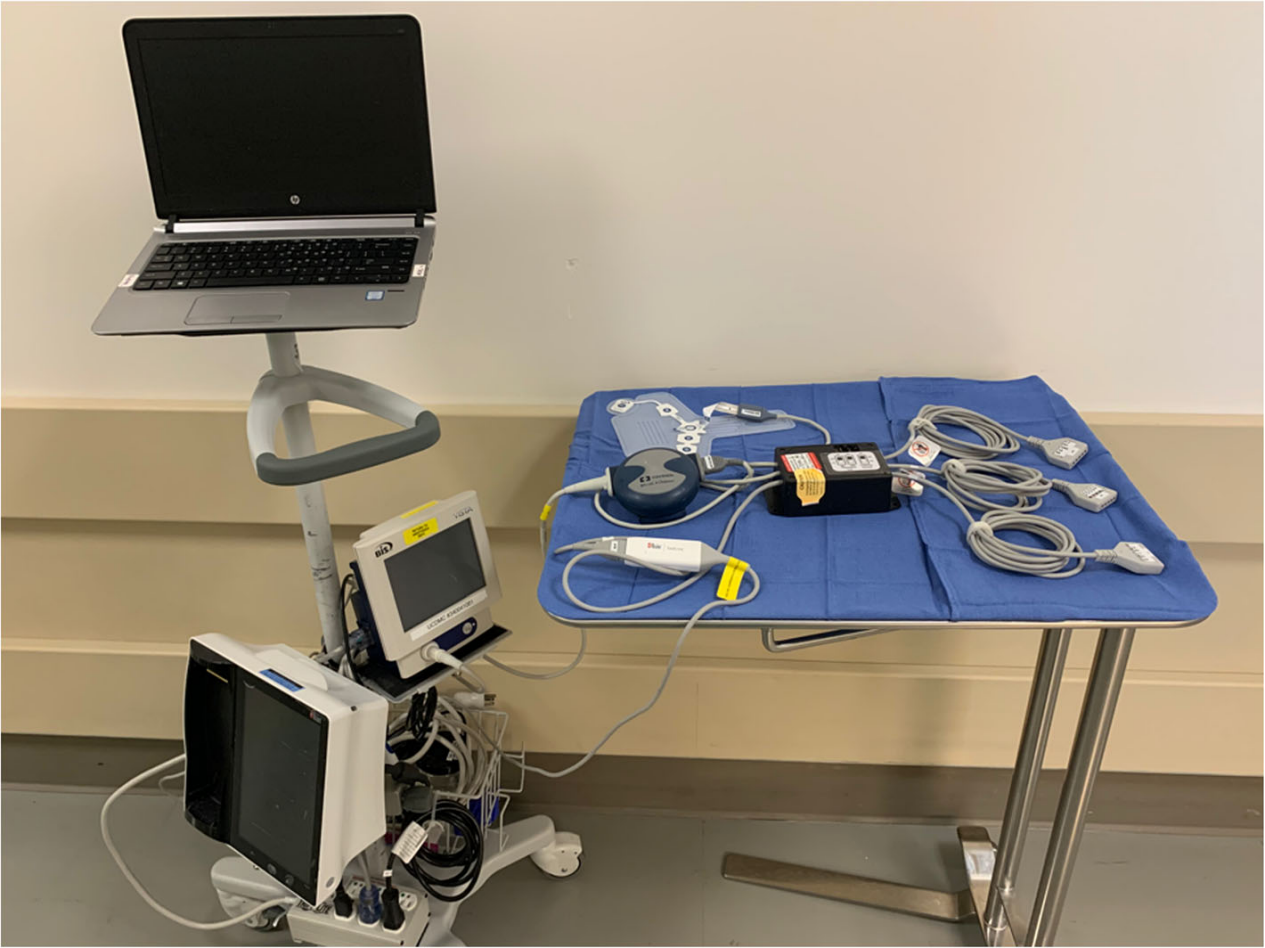
Mobile data collection station showing laptop computer with data collection software; custom interface adapter with ports for connection to sensory electrodes and cables to BIS and SedLine monitors; BIS monitor; and SedLine Root monitor

The patient will then be transported to the operating room where the EEG sensors will be connected to a custom-built passive interface box. This box contains only resistors and wires and no active electronics. Connections extend from this box to the BIS and SedLine modules as demonstrated in Fig. 2. BIS and SedLine data are recorded using Rugloop (2018 version 10.10) and Masimo Automated Data Collection (proprietary, 2017 version 10.4) software, respectively. The data collection system is shown in Fig. 3.

Prior to beginning data collection, each sensor will be checked to ensure that impedance of each channel is less than or equal to 15 kohms (kiloohms). Sensors with an impedance greater than 15 kohms will be permissible only if the EEG waveform is deemed to be of acceptable quality. Unacceptable EEG waveform quality will prompt application of additional gel to reduce impedance or sensor replacement. After a successful impedance test is recorded, impedance testing will be disabled, and data collection initiated.

### Data collection

Data collection will begin with a baseline set of BIS and PSI indices. Once recorded, general anesthesia will be induced with medications and doses chosen by the intraoperative anesthesia care team and documented in the electronic medical record (EMR). The depth of sedation during induction will be measured, at least, every minute using the Modified Observers’ Assessment of Alertness/Sedation (MOAAS) scale until the patient loses the eyelash reflex and achieves a score of 0. Two MOAAS scores can share the same timepoint if the patient achieves a new score less than 1 min from the prior score. The following descriptions of MOAAS scores will be used: 0 does not respond to pain; 1 does not respond to mild prodding or shaking; 2 responds after mild prodding or shaking; 3 responds after calling loudly or repeatedly; 4 responds slowly to voice with normal tone; 5 responds readily to voice with normal tone. Times indicating start of anesthesia induction, endotracheal intubation, surgical incision, and the start of electrocautery will also be recorded. Electronic, time-stamped documentation of intravenous medication doses in the EMR by the anesthesia provider will allow for retrospective review of their effects on raw and processed EEG data by investigators. Similarly, end-tidal concentrations of volatile gases will be automatically recorded in real-time within the patient’s EMR throughout the entire surgery.

Throughout the course of the surgical procedure, timepoints with acute changes of ±20% in blood pressure or heart rate from baseline values will be recorded. At these timepoints, an investigator will question the anesthesia provider regarding the suspected cause for each acute change as “Light Anesthesia”; “Deep Anesthesia”; “Volume Status”; or “Other.” The anesthesia provider will not be limited in responses for “Other.”

**Fig. 4:**
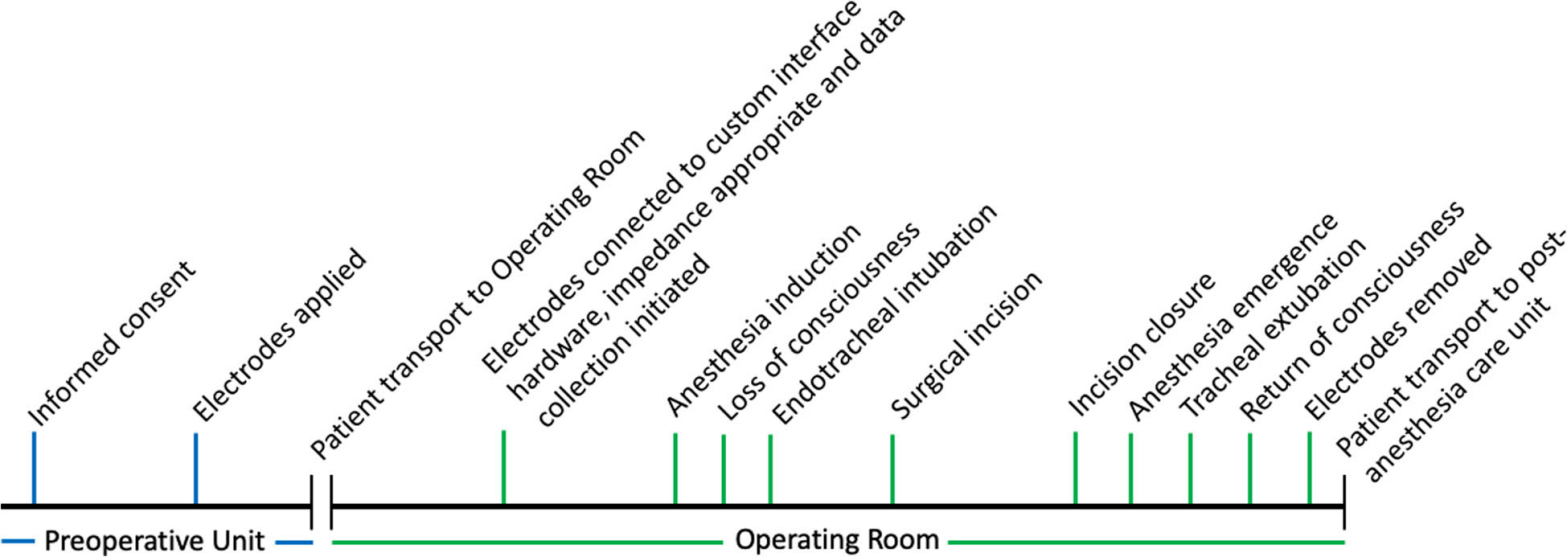
Timeline of Events and Experimental Recordings

At the end of the surgical procedure, timepoints indicating incision closure, emergence from anesthesia, tracheal extubation, and return of consciousness will be recorded. During emergence, MOAAS will be measured every minute until the patient achieves a score greater than or equal to 4. Sensors will be removed prior to patient transport to the post-anesthesia care unit (PACU). A timeline of events and experimental recordings are shown in Fig. 4.

Raw and processed EEG data will be organized according to the patient’s unique study identification number. Record of medication administration will be stored in the EMR and accessible to investigators by searching the patient’s medical record number (MRN). Preoperative patient demographics, vital signs, ethnicity, race, ASA status, description of surgical procedure, pre-existing diseases, surgical position, MOAAS scores, event timepoints, anesthesiologists’ assessments, and general comments will be written on the CRF, which will then be scanned, and electronically stored in a password-protected, encrypted N: drive.

### Monitoring

Data monitoring will occur regularly with and without sponsor involvement to ensure that data collection is accurate and complete. The investigators and sponsor of this protocol will have access to interim analyses and decide to amend the protocol or terminate the trial, if applicable. Furthermore, trial conduct will be regularly audited by the sponsor, and amendments will be communicated with the IRB.

### Primary outcomes

This protocol’s primary endpoint is to establish the concordance, or extent of agreement, between the processed EEG indices from BIS and SedLine monitors and the real-time clinical assessments of anesthetic depth by clinicians during “Deep Anesthesia Events” and “Arousal Events.” Investigators will retrospectively review the EMR to identify interventions made by the anesthesia providers during 10 min surrounding (5 min before and after) periods of burst suppression noted on raw EEG or acute changes in blood pressure or heart rate ± 20% from baseline values. The performace of processed EEG indices surrounding periods of burst suppression on raw EEG will also be analyzed.

Clinically identified possible “Deep Anesthesia Events” are defined as acute decreases in blood pressure or heart rate greater than or equal to 20% from the patient’s preoperative baseline values. The clinical diagnosis of “Deep Anesthesia” coupled with any of the following on the raw EEG, BIS or SedLine monitors indicates agreement: burst suppression on raw EEG, processed EEG index value consistent with a deep anesthesia state per manufacturer recommendations (below 40 for BIS or below 30 for SedLine), or an acute decrease in the processed EEG index by 10 over the same index 5 min prior to the event. The actions of the anesthesia providers will also be examined during these timepoints, in particular the administration of hypnotic (such as propofol or volatile agent) or analgesic medication.

Clinically identified possible “Arousal Events” are defined as acute increases in blood pressure or heart rate greater than or equal to 20% from the patient’s preoperative baseline values. The clinical diagnosis of “Light Anesthesia” coupled with any of the following on the BIS or SedLine monitors indicates agreement: processed EEG index value that signifies inadequate anesthesia per manufacturer recommendations (greater than 60 for BIS or greater than 50 for SedLine), or an acute increase in processed EEG index by 10 over the same index 5 min prior to the event. The actions of the anesthesia providers will also be examined during these timepoints, in particular the administration of hypnotic (such as propofol or volatile agent) or analgesic medication.

### Statistical analysis

The percentage of agreement between assessments of anesthesia depth during clinically identified possible “Deep Anesthesia Events” and “Arousal Events” from BIS and SedLine monitors and clinicians’ assessment will be analyzed with chi-square test. Kappa statistic and the standard error of kappa will also be calculated to account for the degree of agreement expected by chance. All statistical comparisons will be performed using GraphPad Prism version 8.4.3 for Windows, GraphPad Software, San Diego, California USA, (www.graphpad.com).

## Discussion

We present a novel data collection system that will support a prospective, non-blinded, non-randomized study to concurrently compare the performance of processed EEG indices from BIS and SedLine monitors. Although prior studies have demonstrated marginal correlation between the BIS and SedLine monitors with various anesthetic agents, these studies have been limited because they require non-standard sensor locations in order to apply both sensor arrays on the forehead of study subjects [22, 23]. Because not all sensors are positioned according to manufacturer’s instructions, the validity of prior comparisons is compromised. In addition, our inclusion of standardized clinical observations (acute changes in blood pressure or heart rate) in real-time and collection of raw and processed EEG waveforms will allow us to investigate the agreement of BIS and PSI indices across a wide range of anesthetic depth and establish correlations to clinical assessments and medication administration.

We recognize several limitations in our study design. Processed EEG indices may provide support for the clinical evaluation of anesthetic depth or disagree with clinical assessments during periods of arousal or deep anesthesia states. Therefore, a clinician’s decision to modify an anesthetic by administering additional volatile gas, opioid, or other medication may not always be in agreement with real-time brain function monitoring. Furthermore, changes in processed EEG indices may be delayed relative to cardiovascular parameters. Although the intraoperative anesthesia care team will be blinded to the processed EEG indices, the investigators are not blinded but will not report the processed EEG value to the anesthesia clinician at any point during the study. Our study design may be biased by the Hawthorne effect. The intraoperative anesthesia care team may behave differently while being watched by an investigator. In this setting, they may react to changes in blood pressure and heart rate differently when asked about these acute changes by the investigator and initiate interventions sooner or more frequently because of the observations. However, this observation is essential to capture the clinical assessment of possible “Deep Anesthesia Events” or “Arousal Events.” Although changes in cardiovascular parameters (heart rate and blood pressure) by 20% from the preoperative baseline may not always be clinically significant, redefining “Deep Anesthesia Events” and “Arousal Events” with greater absolute changes risks missing data points. Furthermore, this study is not randomized and has no control group. Data regarding associated outcomes such as postoperative delirium, PACU discharge time, accidental awareness, or recall will not be collected. However, this dataset may allow us to further investigate whether these measurable postoperative outcomes are linked to clinician and processed EEG disagreements regarding anesthesia depth, and could provide a focus for future investigation.

Our data may not be representative for all patient groups, particularly pediatric patients and those with severe systemic disease (ASA IV or V). Patients undergoing surgical procedures in the prone or lateral positions are also not represented given the variable contact between the EEG sensors and the patients’ forehead skin in these positions and the inability to reliably troubleshoot these problems. Additionally, interference from electrocautery limits our ability to reliably evaluate processed EEG indices in patients undergoing procedures of the head, neck, and face.

Real-time, simultaneous comparison of brain function monitoring devices using this electronic interface is the only way by which we can confidently and accurately investigate differences in proprietary algorithms. Our study design and custom engineered setup will allow a valid comparison of the BIS and PSI indices. Further research with our design will be necessary given the potential benefits of accurately assessing anesthetic depth.

### Trial status

This study is currently in the recruitment phase. Earlier publication of our study design was delayed so that the electrode sensors, custom-built passive interface box, and data collection system could be appropriately evaluated in clinical practice.

**Fig. 5:**
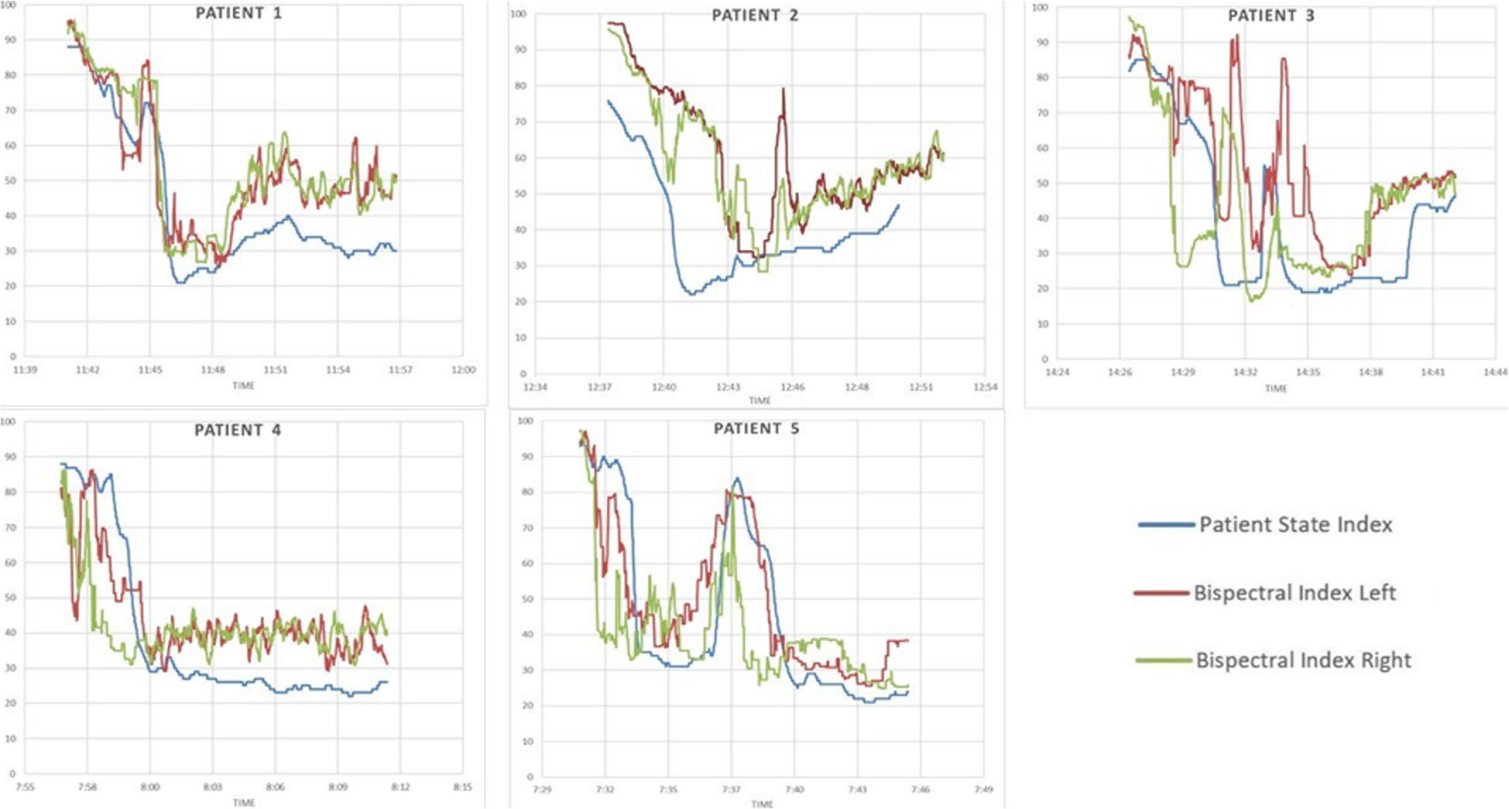
Changes in BIS and PSI indices during general anesthesia induction for 5 different patients

Sample recordings from 5 patients during general anesthesia induction utilizing our data collection system are shown in Fig. 5. Overall, BIS and PSI indices appear to change in parallel during general anesthesia induction. However, qualitative differences are apparent in studying these data.

## Data Availability

Available upon request from corresponding author (JJ) at jjones2@ad3.ucdavis.edu.

## Abbreviations

ASA: American Society of Anesthesiologists
ADC: Automated data collection
GND: BIS ground channel
BIS: Bispectral index
CRF: Case report form
CT: Common reference channel
ECG: Electrocardiogram
EEG: Electroencephalogram
EMG: Electromyography
EMR: Electronic Medical Record
CB: Ground channel
kohms: Kiloohms
LE: Left eye
LT: Left temple
MOAAS: Modified Observers’ Assessment of Alertness/Sedation
MRN: Medical Record Number
PSI: Patient state index
PACU: Post-anesthesia care unit
RE: Right eye
RT: Right temple
